# Social Inequalities in Internet Use, Digital Skills, and Health Literacy Among Older Caregivers

**DOI:** 10.1093/geroni/igaf122.1144

**Published:** 2025-12-31

**Authors:** Roosa-Maria Savela, Tarja Välimäki, Su-I Hou

**Affiliations:** University of Turku, Turku, Varsinais-Suomi, Finland; University of Eastern Finland, Kuopio, Pohjois-Savo, Finland; University of Central Florida, Orlando, Florida, United States

## Abstract

**Introduction:**

Little is known about social inequalities in older adults’ digital skills and Internet use, which may also interact with health literacy.

**Methods:**

The issue is assessed with the ninth wave of the quantitative cross-sectional panel data from the Survey of Health, Ageing and Retirement in Europe (SHARE). Data were analyzed using a Chi-squared test, and regression models were adjusted for demographic factors focusing on inequalities in Internet use, digital skills, and health literacy among older adults with and without informal care tasks.

**Results:**

Informal caregivers reported lower digital skills (63% vs. 56%, p < 0.001) and were less likely to use the Internet in the past week (40% vs. 35%, p < 0.001) than non-caregivers. Informal caregivers were also more likely to constantly than never need help interpreting written instructions from healthcare (Odds ratio [OR]=2.03, 95% Confidence Interval [CI]=1.12 to 3.67), indicating lower health literacy than non-caregivers. However, older adults who were easily making ends meet reported higher health literacy (93% vs. 65%, p < 0.001) or more likely good to excellent digital skills (56% vs .19% p < 0.001) than those who were less financially secure. Conversely, older adults who were easily making ends meet (OR 2.06, 95% CI 1.75 to 2.43) and those with high education levels (OR 3.04, 95% CI 2.72 to 3.40) were most likely to use the Internet in the past week.

**Discussion:**

Higher socioeconomic and non-caregiver status may predict better outcomes in Internet use, digital skills, and health literacy, emphasizing the need to address social inequalities.

